# The Immune Landscape of Human Pancreatic Ductal Carcinoma: Key Players, Clinical Implications, and Challenges

**DOI:** 10.3390/cancers14040995

**Published:** 2022-02-16

**Authors:** Marie Muller, Vincent Haghnejad, Marion Schaefer, Guillaume Gauchotte, Bénédicte Caron, Laurent Peyrin-Biroulet, Jean-Pierre Bronowicki, Cindy Neuzillet, Anthony Lopez

**Affiliations:** 1 Department of Gastroenterology, Nancy University Hospital, University of Lorraine, 54500 Vandœuvre-lès-Nancy, France; v.haghnejad@chru-nancy.fr (V.H.); m.schaefer@chru-nancy.fr (M.S.); b.caron@chru-nancy.fr (B.C.); l.peyrin-biroulet@chru-nancy.fr (L.P.-B.); jp.bronowicki@chru-nancy.fr (J.-P.B.); a.lopez@chru-nancy.fr (A.L.); 2 Department of Pathology, Nancy University Hospital, University of Lorraine, 54500 Vandœuvre-lès-Nancy, France; g.gauchotte@chru-nancy.fr; 3 INSERM U1256, NGERE, Faculty of Medicine, University of Lorraine, 54500 Vandœuvre-lès-Nancy, France; 4 Medical Oncology Department, Curie Institute, Versailles Saint-Quentin University (UVQ), Paris Saclay University, 92064 Saint-Cloud, France; cindy.neuzillet@curie.fr

**Keywords:** pancreatic ductal adenocarcinoma, tumoral microenvironment, immune cells, desmoplastic stroma

## Abstract

**Simple Summary:**

Pancreatic ductal adenocarcinoma (PDAC) is one of the most aggressive and deadliest cancer worldwide, with an overall survival (OS) rate, all stages combined, of still <10% at 5 years. For most patients with unresectable or recurrent PDAC, few therapeutic options are available with limited efficacy (median OS ≤ 12 months). Moreover, immune checkpoint inhibitors that have been tested so far in PDAC failed to improve patient survival. This resistance of PDAC to therapies is, in part, related to the dense tumor stroma, which creates a mechanical barrier around the tumor cells and generates high interstitial pressure, preventing appropriate vascularization and thus limiting exposure to treatments. In that PDAC tumoral microenvironment, the inflammatory cell infiltrates are unbalanced toward an immunosuppressive over pro-inflammatory phenotype. Therefore, there is an urgent need to better define the composition of PDAC stroma and key immune players in order to identify new therapeutic targets and strategies.

**Abstract:**

Pancreatic ductal adenocarcinoma (PDAC) is one of the most aggressive and deadliest cancer worldwide with an overall survival rate, all stages combined, of still <10% at 5 years. The poor prognosis is attributed to challenges in early detection, a low opportunity for radical resection, limited response to chemotherapy, radiotherapy, and resistance to immune therapy. Moreover, pancreatic tumoral cells are surrounded by an abundant desmoplastic stroma, which is responsible for creating a mechanical barrier, preventing appropriate vascularization and leading to poor immune cell infiltration. Accumulated evidence suggests that PDAC is impaired with multiple “immune defects”, including a lack of high-quality effector cells (CD4, CD8 T cells, dendritic cells), barriers to effector cell infiltration due to that desmoplastic reaction, and a dominance of immune cells such as regulatory T cells, myeloid-derived suppressor cells, and M2 macrophages, resulting in an immunosuppressive tumor microenvironment (TME). Although recent studies have brought new insights into PDAC immune TME, its understanding remains not fully elucidated. Further studies are required for a better understanding of human PDAC immune TME, which might help to develop potent new therapeutic strategies by correcting these immune defects with the hope to unlock the resistance to (immune) therapy. In this review, we describe the main effector immune cells and immunosuppressive actors involved in human PDAC TME, as well as their implications as potential biomarkers and therapeutic targets.

## 1. Introduction

Pancreatic ductal adenocarcinoma (PDAC) is one of the most aggressive and deadliest cancer worldwide, with a steadily increasing incidence, especially in industrialized countries [1,2]. Projections indicate that PDAC will be a leading cause of cancer mortality in Europe [1] and the United States [3] by 2030–2040. This increase in incidence is not fully explained by “classical” PDAC risk factors (i.e., age, tobacco smoking, diabetes mellitus, obesity/overweight, chronic pancreatitis, and genetics/family history). It also remains a major challenge in digestive oncology due to its high lethality despite many explored therapeutic strategies, with overall survival (OS) rate, all stages combined, of still <10% at 5 years [4]. Several well-established factors may account for this poor prognosis. First, curative surgical management of PDAC is not possible at the time of diagnosis for 85% of patients, owing to vascular invasion (locally advanced disease) or distant metastases [5]. Moreover, even when resectable, PDAC recurrence is often observed within the first two years after complete resection due to micrometastatic disease (present in 90% of patients), justifying systematic administration of adjuvant therapy in all resected patients [6]. For patients with unresectable or recurrent PDAC, therapeutic options are mainly based on combined chemotherapy regimens (FOLFIRINOX or Gemcitabine + nab-paclitaxel as first-line therapies) with limited efficacy (median OS ≤ 12 months) [6,7]. Finally, all immune therapies that have been tested so far in PDAC failed to improve patient survival. This resistance of PDAC to conventional therapies is, in part, related to the desmoplastic reaction, also known as stroma, that characterizes these tumors [8]. Indeed, this histopathological hallmark of PDAC is found in both primary PDAC and its metastases [9]. PDAC stroma is composed of extracellular matrix (ECM) and several cell types including cancer-associated fibroblasts (CAFs, which are responsible for ECM production) and inflammatory immune cells [10]. The dense ECM deposits create a mechanical barrier around the tumor cells and generate high interstitial pressure, preventing appropriate vascularization and thus limiting exposure to chemotherapy, leading to a hypoxic tumor microenvironment (TME) [11]. In this hypoxic TME, the inflammatory cell infiltrates are unbalanced toward an immunosuppressive over pro-inflammatory phenotype, with a high prevalence of myeloid-derived suppressor cells (MDSCs), M2 polarized macrophages, and regulatory T cells (Tregs), over M1 macrophages, dendritic cells, and effector CD4+ and CD8^+^ T lymphocytes [12].

Overall, PDACs are immune deserts or immune-excluded tumors, which can explain why immune checkpoint inhibitors (ICIs) and other immune approaches still did not show efficacy in PDAC [13].

In this review, we summarize current acquired knowledge about the immune landscape in human PDAC and its impact on survival and therapy in patients with PDAC.

## 2. The Key Role of Cancer-Associated Fibroblasts in PDAC Immune Macroenvironment

CAFs, through their ECM production and interactions with tumor and other stromal cells, play the central role of “orchestrators” of the PDAC microenvironment. Several cells of origin for CAFs have been proposed, including pancreatic stellate cells (PSCs). PSCs are a unique type of resident pancreatic cells that exist in normal pancreatic tissue in a quiescent form [14]. PSCs are engaged in several homeostatic roles under physiological conditions [15] (i.e., vitamin A storage, phagocytosis, immunity, and stimulation of amylase secretion from the exocrine pancreas) and also under pathological states as pancreatitis or PDAC in which they become activated [16,17]. Upon activation, they acquire a myofibroblastic, contractile phenotype (expressing alpha-smooth actin) and produce soluble factors (growth factors, cytokines) and ECM components [18].

**Figure 1:**
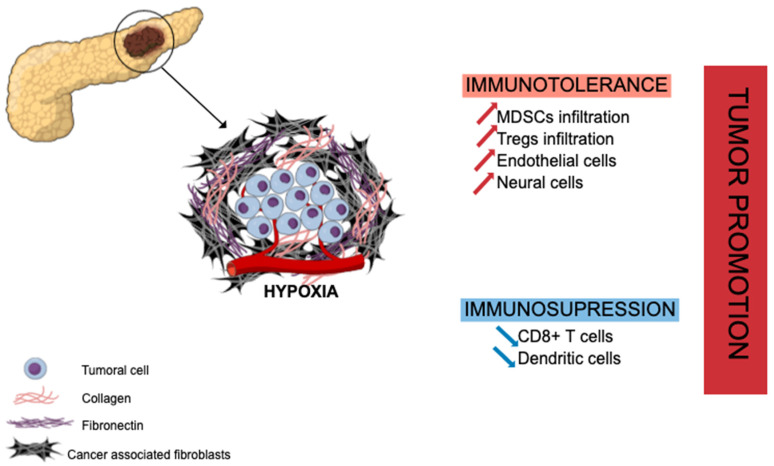
Extracellular matrix in pancreatic ductal adenocarcinoma and tumor growth promotion. Abundant stroma is mainly composed of cancer-associated fibroblasts (CAFs) and collagenous proteins leading to hypoxia, immune evasion, and immunotolerant state through recruitment of immunosuppressive cells (MDSCs, Tregs) and suppressive effect on effector immune cells (CD8^+^ T cells, dendritic cells). MDSCs: myeloid-derived suppressor cells; Tregs: regulatory T cells.

PDAC has a unique fibrotic TME, also known as desmoplastic stroma, illustrated in Figure 1 hereafter. This stroma is abundant (often representing the majority of tumor volume) and mainly composed of ECM proteins produced by CAFs (e.g., collagen, laminin, fibronectin) along with non-collagenous molecules (e.g., glycoprotein, proteoglycans, glycosaminoglycans, hyaluronic acid) [19]. This ECM enclosed various cell types, including different immune cells such as lymphocytes (rare), macrophages, mast cells, and myeloid-derived suppressor cells (MDSCs), along with endothelial and neuronal cells, to set up a growth permissive and immunotolerant microenvironment for pre-neoplastic pancreatic cells [20] (PanINs) and for pancreatic tumor cells [21]. The ECM is a dense meshwork of structural proteins, adaptor proteins, proteoglycans, and enzymes found in all tissues, where it provides biochemical and structural support for tissue homeostasis. In PDAC, there is a marked increase in the deposition of ECM [22]. Specifically, type I, III, and IV collagens are the main structural proteins constituting PDAC ECM. Pancreatic cancer cells induce desmoplastic responses within the tumor stroma by stimulating stromal fibroblasts to upregulate the expression of collagen family proteins and fibronectin in a paracrine manner [23]. Despite the hypoxia and hyaluronan-induced development of desmoplastic stroma, PDAC TME is composed of several immune cell populations that we will describe hereinafter. At early stages, effector cells such as natural killer (NK) cells, CD8^+^ T cells, and CD4^+^ T cells can be present and activated. Nevertheless, during the selection of resistant tumor cells and the development of the escape mechanism, PDAC TME induces the recruitment of monocytes and neutrophils, which then have acquired an anti-inflammatory phenotype [24]. The transformation of pro-inflammatory to anti-inflammatory in the TME increases the tumor growth and angiogenesis and correlates with poor survival, as well as systemic immunosuppression and malnutrition in patients.

In PDAC, CAFs are strongly involved in the mechanism of immune evasion and the immunotolerant state toward pancreatic tumor cells through the cytokine-induced (IL-6, IL-11, TGF-β signaling) recruitment of immunosuppressive cells such as regulatory T cells (Treg cells) [21], myeloid-derived suppressor cells (MDSCs) [25,26] or programed death 1 ligand (PD-L1) mainly expressed on T cells [27]. CAFs and the hypoxia that they generate are also associated with a suppressive effect on effector immune cells as CD8^+^ T cells [28] or dendritic cells [29,30], hampering their infiltration into PDAC tumor areas. However, recent studies have highlighted that PDAC CAFs are heterogeneous, some of them being pro- and other anti-tumoral [31,32]. Several classifications have been proposed [33]. Therefore, a better understanding of CAF molecular and functional heterogeneity is required.

CAFs are involved in a growth-supportive TME in PDAC, especially through their complex interactions with immune cells, leading to an immunotolerant and unique immunosuppressive state. From a therapeutic perspective, despite the preclinical success of several agents targeting ECM (e.g., metalloproteinases inhibitors, hyaluronidase (PEGPH20)), this strategy failed to show any clinical activity in patients with advanced-stage PDAC [11]. A better understanding of those complex interactions might be useful to develop more effective immunotherapies against PDAC. Targeting the ECM alone is not effective in pancreatic cancer.

## 3. Effector Immune Cells in PDAC

**Figure 2:**
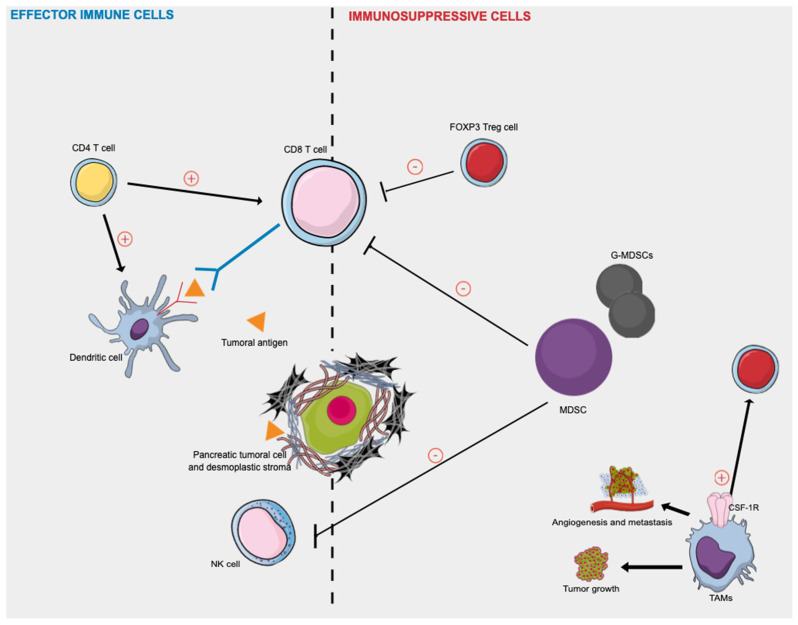
Effector immune cells and immunosuppressive cells in human pancreatic cancer. Activated CD8^+^ T cells attack tumor cells presenting tumor-associated antigens peptides on their surface, as dendritic cells. CD4^+^ T cells target tumor cells either directly by eliminating tumor cells through cytolytic mechanisms or indirectly by modulating the TME. Tregs and MDSCs cells play immunosuppressive roles in inhibiting the immune response against PDAC cells by directly inhibiting the anti-tumor functions of T and NK cells. The TAM phenotype is a consequence of the continuous presence of growth factors such as colony-stimulating factor-1 (CSF1) and its receptor (CSF-1R). TAMs release various growth factors and cytokines and promote tumor cell invasion, induce angiogenesis, suppress antitumor immunity, and facilitate tumor cell metastasis. CSF-1R: colony-stimulating factor-1 receptor; MDSC: myeloid-derived suppressor cells; NK: natural killer; TAMs = tumor-associated macrophages; Treg = regulatory T cell.

Figure 2 presented afterward summarizes the key effector immune cells and immunosuppressive actors in human PDAC.

### 3.1. Tumor-Infiltrating Lymphocytes: CD8^+^ T and CD4^+^ T Cells

Tumor-infiltrating lymphocytes (TILs) are often observed in resected cancer tissue and are believed to participate in the host immune response against cancer [34,35]. They can be organized in tertiary lymphoid structures, which have been shown to have a positive prognostic impact [36].

Some TILs might be involved in restraining tumor development and progression. Intratumoral T cell infiltrates were shown to form an independent positive prognostic marker in colorectal cancer (CRC) [37] and also PDAC [38]. It could predict patients’ survival more efficiently than the TNM staging system (Immunoscore staging system) [37]. The most studied TILs are CD8^+^ T cells and CD4^+^ T cells, presented in Figure 2.

CD8^+^ T cells (cytotoxic T cells) are believed to identify cancer as a foreign body in a major histocompatibility complex class I-restricted manner [39]. Activated CD8^+^ T cells attack tumor cells presenting tumor-associated antigens peptides on their surface [39,40]. High levels of tumor infiltration with especially CD8^+^ lymphocytes are a characteristic of an immunogenically hot tumor, which better respond to immune checkpoint inhibitors [41].

CD4^+^ T cells play major roles in the host immune response against cancer and are required for efficacious antitumor immunity [42]. CD4^+^ T cells can target tumor cells in various ways, either directly by eliminating tumor cells through cytolytic mechanisms (perforin/granzyme B-dependent manner) or indirectly by modulating the TME [43].

In PDAC, total T lymphocytes (CD3) have been investigated in several studies [44,45,46,47,48,49,50,51,52,53,54,55,56,57,58,59,60]. It is estimated that >80% of the CD3^+^ T cells are either CD4^+^ or CD8^+^ in tumoral pancreatic tissues [55]. Among those studies, CD3^+^ TIL rate was correlated to patient survival in 10 of them. High CD3^+^ TIL rate was significantly associated with better disease-free survival (DFS) and/or OS in 6 studies [45,46,51,53,58,59]. A summary of the described studies assessing CD3^+^ cells in PDAC is reported in Table 1.

CD4^+^ and/or CD8^+^ TILs assessment have also been the topic of several studies in PDAC [35,39,44,45,46,48,50,52,54,56,57,58,59,61,62,63,64,65,66,67,68,69,70,71]. Indeed, CD8^+^ T cells are the most studied T effectors in PDAC. Considerable lines of evidence suggest that a high CD8^+^ T cells infiltration in PDAC tumoral tissue is significantly associated with a better DFS and/or OS [39,50,54,56,58,59,62,63,65,67,68,69,70,71]. In the largest series of 165 patients operated on for PDAC [56], a high number of CD8^+^ lymphocytes was significantly and independently associated with longer DFS and OS in the overall study population. Median DFS/OS were 7.4/18.1 months for patients with a low number of CD8^+^ intratumoral lymphocytes (defined as ≤ 42 per 1 mm tissue core) and 12.7/25.2 months for patients with high CD8^+^ TILs numbers [56]. A summary of the described studies assessing CD4^+^ and/or CD8^+^ cells is reported in Table 2.

Those data suggest that TILs, especially CD4^+^ and CD8^+^ T cells effectors, are interesting and easily measurable indicators of PDAC immune TME. However, most PDACs are not infiltrated by cytotoxic CD8^+^ T cells. Several factors may be involved: low immunogenicity, T cell exclusion by other immune cells (immunosuppressive Treg, MDSCs). Moreover, most “hot” PDAC tumors in terms of CD8^+^ T cells are also infiltrated by macrophages, which are able to inhibit their cytotoxic activity [13].

### 3.2. Dendritic Cells

Dendritic cells (DCs, S100+ cells) are a diverse group of specialized antigen-presenting cells (APCs) [72]. DCs are able to induce antigen-specific immune responses and play major roles in tumor immunity, especially myeloid DCs (mDCs, CD11b+ cells). They are able to recognize tumor-associated antigens in tumor tissue and generate tumor-specific immunity, as described in Figure 2 [73]. DCs are rare in PDAC TME and are mainly located in the stroma peripheral to the tumor [74]. For the last decade, DCs have been a subject of interest in the study of PDAC immune TME [75] and have emerged as the immune cells of choice for the development of a vaccine against PDAC [76]. It was shown that circulating DCs or high-level DCs in PDAC tissues were significantly associated with better OS, whatever PDAC stage at diagnosis (resectable or metastatic disease) [77,78,79]. In a group of 42 patients with resected PDAC, Yamamoto et al. revealed that the OS rate in the high tissue-DCs group (DCs ≥ 3.5) was significantly longer than that in the low tissue-DCs group (*p* = 0.038, 1/2/3 year OS: 91%/70%/35% versus 67%/41%/27%) [79]. It was also demonstrated that the number of PDAC-infiltrating DCs was significantly higher in PDAC with CD4^+^ and CD8^+^ TILs infiltration [39]. These data suggest that if both CD4^+^ and CD8^+^ TILs are found in PDAC tissue, all key components of an antitumor immune response are present, from the presentation of tumor-associated antigens by DCs to the interaction of helper T cells and cytotoxic T cells to attack PDAC tumor cells [39].

PDAC is characterized by the reduced number and function of DCs, which impact antigen presentation and contribute to immune tolerance. As DCs are the most potent professional antigen-presenting cells, several ongoing studies use DC-based vaccines or agents promoting DC maturation (e.g., CD40 agonists) as potential immune therapies in PDAC.

### 3.3. Innate Lymphoid Cells

Innate lymphoid cells (ILCs) represent a heterogeneous group of cells that derive from common lymphoid progenitors in the bone marrow [80]. Five major groups of ILCs have been defined on the basis of their cytokine production patterns and developmental transcription factor requirements: natural killer (NK) cells, group 1 ILCs (ILC1s), group 2 ILCs (ILC2s), group 3 ILCs (ILC3s), and lymphoid tissue-inducer (LTi) cells [80].

Natural killer cells (NK cells, CD56+, CD3−) are large granular lymphocytes that are key components of the innate immune system [81]. The role of NK cells remains poorly understood, compared with other lymphocytes, especially T and B cells. Activation of NK cells mainly relies on the balance of signals received from inhibitory and activating cell surface receptors. In different cancer types, NK cells demonstrated potent antitumoral cytotoxicity, even if they appear to be highly heterogeneous between cancer types [82,83,84]. In human PDAC, few data are available but suggest antitumor activity. Indeed, to our knowledge, only one positive correlation was demonstrated between the percentage of NK cells in peripheral blood and recurrence-free survival in a few patients (*n* = 19) with resectable PDAC [85]. Patients who exhibited high NK levels before surgery significantly had later disease recurrence.

ILC1s, ILC2s, and ILC3s resemble the corresponding T helper cell subsets (T helper 1 (TH1), TH2, and TH17 cells, respectively) and produce cytokines that shape both innate and adaptive immune responses [80]. Recently, it was demonstrated that ILC2s infiltrate human PDAC to activate tissue-specific tumor immunity [86]. Using microarrays from formalin-fixed, paraffin-embedded from 45 PDAC short-term survivors (OS < 3 years) and from formalin-fixed, paraffin-embedded from 51 PDAC long-term survivors (OS ≥ 3 years), authors highlighted the fact that tumor ICL2 (TILC2s) were enriched in rare long-term PDAC survivors with “hot” tumors (activated CD8^+^ T-cell-enriched types) when compared with short-term survivors with “cold” tumors [86]. Moreover, higher TILC2 frequencies correlated with longer survival [86].

Although ILC (and more specifically ILC2s) could emerge as novel anticancer immune cells and potential targets for PDAC immunotherapy, their prognostic and predictive potential remains to be determined. Further investigations are needed to better understand ILC(2) role in PDAC and to confirm ILC2s as potential anticancer immune cells for PDAC immunotherapy.

From a therapeutic perspective, enhancing T-cell effector functions could be a promising therapeutic approach against human PDAC. Recent advances in the field of antigen identification, T-cell biology, and gene therapy have enabled the development of promising strategies such as chimeric antigen receptor T-cell (CAR-T cell) therapy and T-cell receptor T-cell (TCR-T) therapy to redirect T-cell antigen specificity and enhance T-cell function (adoptive T-cell therapy (ATCT)). However, ATCT remains unproven in human PDAC.

## 4. Immunosuppressive TME in PDAC

Figure 2 summarizes the key effector immune cells and immunosuppressive actors in human PDAC.

### 4.1. Regulatory T Cells

Regulatory T cells (Tregs) are major players in tumor immune suppression in PDAC. They can be identified based on forkhead box protein 3 (FOXP3) protein expression and high levels of interleukin-2 receptor alpha chain CD25 through immunohistochemistry in tumor tissues. There is abundant evidence to suggest that Tregs represent the main obstacle to successful tumor immunotherapy [87]. Indeed, Tregs accumulate in tumors and the peripheral blood of patients with cancer. Tregs are recruited to tumors sites where they suppress antitumor cytotoxic response by binding to DCs and preventing them from activating CD8^+^ T cells [88], as summarized in Figure 2. It has been demonstrated that Tregs contribute to inhibiting the immune response against PDAC cells, from the premalignant stage to invasive PDAC [89]. Moreover, in PDAC, a high prevalence of Tregs seems to be associated with poor prognosis and poor PDAC differentiation [68]. Treg infiltration in PDAC has been well studied since the 2000s. Several studies demonstrated that Tregs FOXP3+ immunostaining could be used as a prognostic biomarker and that it could be a crucial determinant of immunosuppressive TME in PDAC [90]. High Treg infiltrates in PDAC tissues were significantly associated with worse survival rates in PDAC [50,91] and conversely, low Tregs infiltrate rates were associated with better survival [68,70,71]. 

Increased peritumoral FOXP3+T-cell (Tregs cells) density is identified as an independent adverse prognostic factor in PDAC [50].

### 4.2. Myeloid-Derived Suppressor Cells

Myeloid-derived suppressor cells (MDSCs) are defined as a heterogeneous population of immature myeloid cells (CD15+, CD11b+) that accumulate during chronic inflammatory conditions such as cancer [92]. They have critical roles in the TME through immunosuppression [93], as they can directly inhibit the antitumor functions of T and NK cells (Figure 2). Their critical implication in different cancer types has been demonstrated [94,95,96]. MDSCs can be characterized into two major types—monocytic MDSCs (M-MDSCs, CD66b−) and granulocytic MDSCs (G-MDSCs, CD15+, CD66b+). Several CXC families of chemokines have been attributed major roles in MDSC recruitment. In human PDAC, it was demonstrated that high levels of CXCL5 correlate with both tumor-infiltrating CD15+ granulocytes and worse prognosis in patients with PDAC [97,98]. Indeed, in a cohort of 153 patients with resected PDAC, mean OS was significantly higher in the low CXCL5 expression group (immunostaining ≤ 50%) in comparison with the high CXCL5-expression group (immunostaining > 50%) (64.0 months versus 38.5 months, respectively) [97].

Moreover, interleukin-6 (IL-6) has been associated with the enrichment of immunosuppressive MDSCs in several tumor types [92]. The role of MDSCs in human pancreatic TME has also been recently explored [99,100]. In patients with PDAC, it was shown that the plasmatic level of circulating MDSCs was correlated with the stage of the patient’s disease [100,101]. Comparing cancer-free controls and patients with PDAC, Porembka et al. demonstrated that normal pancreatic tissue was free of MDSC infiltration, whereas patients with PDAC have significantly increased levels of MDSCs in the bone marrow and peripheral blood, which are avidly recruited to the primary PDAC sites [100]. MDSC mobilization was significantly most notable in patients with metastatic disease when compared with both normal controls and patients with resectable pancreatic cancer (respectively, 68.2% ± 3.6% versus 37.6% ± 3.6% versus 57.3% ± 3.5; *p* < 0.05 %) [100].

In addition to their main role in the antitumor response, MDSCs might be able to influence the production of Tregs, leading to cancer immunotolerance [102].

Although further studies are needed to confirm those data, MDSCs might be useful markers for PDAC detection and/or progression. They also represent potential therapeutic targets in PDAC oncogenesis.

### 4.3. B Cells

B cells (CD20+) are among important components of the immune infiltrate in solid tumors and are emerging as critical regulators of cancer progression [103]. B cells are known to critically regulate T-cell immune responses, but their role in cancer remains controversial.

One study investigated the role of B cells in the tumoral microenvironment of human PDAC [48]. In that study, the authors demonstrated first of all that human PDAC tissues showed considerable infiltration of CD20+ B lymphocytes, unlike normal pancreatic tissue. Moreover, B cells’ location in PDAC seems to display distinct spatial organization in two histologically distinct compartments: either in ectopic lymph node-like structures (or tertiary lymphoid tissue, B-TLT) [104] or interspersed at the tumor–stroma interface (B-TIL) [48]. Among the 104 PDAC tissue specimens they studied, they revealed that B cells were independently associated with prognosis. However, their prognostic value diverged according to their spatial distribution in the tissue [48]. Indeed, B-TLT were associated with better prognosis (OR = 0.24; 95% CI (0.08–0.71); *p* = 0.010), while B-TIL were associated to worse prognosis (OR = 2.56; 95% CI (0.91–7.23); *p* = 0.07) [48]. This result highlights B cells as prognostic variables in human PDAC and suggests that the influence of B cells on tumor progression changes whether they are confined within lymphoid tissue or are scattered at the tumor–stroma interface.

In PDAC, B cell behaviors seem to be tightly linked to their topological organization within the microenvironment. Further research is needed to better understand their role in PDAC oncogenesis and pancreatic cancer progression.

### 4.4. Tumor-Associated Macrophages 

Macrophages are among the most prominent tumor-associated noncancer cell type in the TME, known as tumor-associated macrophages (TAMs). Current evidence suggests that TAMs engage in complex network interactions with cancer stem cells, cancer cells, endothelial cells, fibroblasts, T cells, B cells, and NK cells, as presented in Figure 2 [105]. TAMs receive signals from diverse cells within the tumor microenvironment and release various growth factors and cytokines and promote tumor cell invasion, induce angiogenesis, suppress antitumor immunity, and facilitate tumor cell metastasis [106]. TAMs are classically classified into two subtypes: M1-polarized macrophages and M2-polarized macrophages, even though this classification is debated. M1-polarized macrophages are characterized by a pro-inflammatory phenotype [106] (i.e., enhanced expression and release of IL-1β, TNF-α, IL-6, or IL-12), whereas M2-polarized macrophages (CD204/206; CD163) exhibit anti-inflammatory properties [106] (i.e., enhanced expression and secretion of immunosuppressive cytokines such as TGF-β1 and IL-10). TAM expression has been studied in three different studies in human PDAC. Kurahara et al. first described an elevated incidence of TAMs, in particular with an M2-polarized phenotype in human PDAC tissues, correlating with increased nodal lymphangiogenesis and poor prognosis [23,107]. Based on tissue specimens from 40 patients with resected PDAC, the authors showed that the nodal lymphatic vessel density had a strong association with the M2-polarized TAM density in the regional lymph nodes (*p* < 0.0001) [23]. They also demonstrated that the 5-year survival rate in the low CD163/204 group and the high CD163/204 group was 28.8% and 5.0%, respectively, with the prognosis significantly poorer in the high CD163/204 group, compared with the low CD163/204 group [107]. More recently, the same observation was made based on 112 cases of surgically resected PDACs [108]. Long-term survival patients (OS ≥ 60 months) display significantly lower densities CD68^+^ cells (total macrophages) and CD163^+^ (M2) macrophages than short-term survival cases (OS < 60 months; *p* = < 0.0001, and < 0.0001, respectively) [108].

From a therapeutic perspective, neutralizing immunosuppressive cells, which are major components of human PDAC TME, could be a potential therapeutic strategy by removing inhibitory factors that constrain T-cell responses. Unfortunately, a phase II clinical study evaluating the inhibition of colony-stimulating factor-1 receptor (CSF-1R, cabiralizumab, M2-like macrophage/TAMs inhibition) with nivolumab and chemotherapy in advanced PDAC patients did not improve progression-free survival, compared with chemotherapy alone (NCT03336216).

Those results highlight the crucial role of tumor-associated macrophages (TAMs) in human PDAC. Such observations could bring new therapeutic hopes, especially in tumors with high infiltration of immunosuppressive TAMs, owing to macrophage targeted drugs (e.g., anti-CSF1R, anti-CXCR2/CCR2), but their activity is hampered by “rescue” secretion of overlapping cytokines.

## 5. Discussion

PDAC TME is a complex network that is still being actively explored. At the immune level, it corresponds to the interactions between different effectors whose functions are still poorly understood, although several studies have been carried out to demystify it, in order to find new therapeutic approaches.

In addition to tumor cell-intrinsic mechanisms, different extrinsic factors may impact the human PDAC TME through immunomodulation. One of the most studied factors and rapidly evolving fields is the microbiome, which also drives the tolerogenic innate and adaptive immune programs in PDAC [109]. The species of bacteria found in human PDAC are distinct from those in the gut, with a greater relative abundance of *Proteobacteria* and reduced α-diversity and richness [109]. Lower intratumoral microbial diversity has been linked to reduced survival, whereas high bacterial diversity correlates with long-term survival in PDAC [110]. Moreover, an intratumoral microbiome signature highly predictive of long-term survival (>5 years) was developed and validated by Riquelme et al. (Pseudoxanthomonas–Streptomyces–Saccharopolyspora–Bacillus clausii) [110]. The microbiome in primary PDAC may exert potent suppressive influences on the inflammatory TME, establishing the tumor-promoting inflammatory program in PDAC by activating the expansion of MDSCs and anti-inflammatory M2-like tumor-associated macrophages (TAMs). These tolerogenic innate immune cells promote the differentiation of suppressive populations of CD4+ T cells and prevent the expansion of cytotoxic CD8^+^ T cells [109]. This suggests that microbiome manipulation might have an excellent potential to overcome the current lack of an effective treatment for chemo-resistant PDAC. Additionally, microbiome manipulation (e.g., fecal microbiota transplantation (FMT)) has been shown to favorably affect the response of PDAC tumors to immunotherapy in animal models [111]. In those animal models, FMT was able to differentially modulate the intratumor microbiome diversity and immune infiltration, with an increase in cytotoxic T cells and a decrease in MDSCs with feces from long-term survivors [110]. Some studies have also linked fungi to the development of PDAC and need further research [112].

Future research efforts are needed to better understand the function and phylogeny of microbes in PDAC using more comprehensive approaches and develop innovative therapeutic strategies. Further studies are also required to determine how microbiota impacts chemotherapy and immunotherapy, in order to generate novel and personalized therapeutic approaches for PDAC patients. Other extrinsic factors as PDAC virome are still under exploration, as well as the role of antibiotics/fungal ablation, chemotherapies, inflammatory pathologies (e.g., pancreatitis, obesity), and physical activity.

Another major difficulty is that human PDAC TME is dynamic and changes with the different stages of pancreatic oncogenesis [113]. This immune landscape evolution has been well described during the progression of intraductal papillary mucinous neoplasm (IPMN) to invasive PDAC in humans [113]. Pancreatic immune TME evolves from a diverse T-cell mixture, comprising CD8^+^ T cells, T helper 1 (Th1, involved in cellular immunity), and Th2 (involved in humoral immunity) as major players combined with Th17 (which produce pro-inflammatory cytokine as IL-17) and Treg cells in low-grade IPMN, to a Treg dominated immunosuppressive state in human invasive PDAC [113]. Moreover, the localization of T cells drastically changes during the natural history of IPMN progression to human invasive PDAC, but this last issue currently remains insufficiently documented [47,57,113]. PDAC TME heterogeneity is currently the subject of intense research. The recently developed method of combining immunity-related genes with clinical characteristics has been applied to predict prognosis, recurrence, and response to gemcitabine-based therapy ± ICI in PDAC. This might be a practical predictive tool to identify PDAC subtype benefitting from gemcitabine-based chemotherapy or potentially responding to PD1/PD-L1 blockade therapy [114,115].

Multiple clinical trials concerning immune-based therapies in PDAC have recently been conducted or are currently underway, so far with limited success. Mostly T-cell effector functions have been targeted in the development of cancer immunotherapies, but endogenous reactive T cells are limited, in quantity and quality, in pancreatic cancer. In PDAC, different immunology-based therapies are under exploration, especially therapeutic vaccines aimed to stimulate T- and B-cell production, and ICI targeting CTLA4, PD1, and programmed death ligand (PDL1). However, ICI targeting these three molecules are mostly ineffective in PDAC. A summary of the main studies assessing ICI in advanced PDAC is reported in Table 3. One explanation for ICI resistance could be the composition of immune cell infiltrate with a PDAC TME having potent immunosuppressive functions, which might interfere with ICI [24]. Therefore, human PDAC is one of the most immune-resistant tumor types, due to immunosuppression and T-cell exclusion from the earliest stages of PDAC development, leading to primary resistance to ICI. Moreover, PDAC is a complex and heterogeneous disease, with diverse clinical phenotypes. Another key of PDAC development and promotion remains in genomics and epigenomics changes, conferring distinct molecular, cellular, and clinical features that determine considerable inter- and intratumoral heterogeneity.

In order to better consider PDAC immune TME as a potential therapeutic target, there is an urgent need to better understand its evolution during PDAC oncogenesis and to gain access to it. The main obstacle remains that desmoplastic reaction around pancreatic tumor islets, which acts similarly to a major physical barrier, limiting access to treatments. Recent treatment strategies focusing on that dense stroma to facilitate the distribution of systemic agents within the tumor microenvironment failed to improve both overall survival and progression-free survival [116]. Combined therapeutic approaches associating TME remodeling with better immunogenicity (e.g., T-cell antigen specificity, enhancing T-cell effector function, immunosuppressive actors’ inhibition), coupled with therapies adapted to the (epi)genetic profile of the tumors, could present a promising perspective in human PDAC.

**Table 3:** Studies assessing immune checkpoint inhibitors in advanced pancreatic ductal adenocarcinoma.

References	Trial Phase	Therapy	Number of Patients	Clinical Outcomes
Laheru et al. 2008 [117]	II	GVAX + cyclophosphamide	50	Median survival: 4.3 months
Royal et al. 2010 [118]	II	Anti CTLA4 antibody (Ipilimumab)	27	No objective response
Brahmer et al. 2012 [119]	I (NCT00729664)	Anti PD-L1 antibody (Nivolumab)	14	No objective response
Le et al. 2013 [120]	Ib (NCT00836407)	Ipilimumab + GVAX	15	Median OS: 5.7 months
Le et al. 2015 [121]	II (NCT01417000)	GVAX + cyclophosphamide	61	Median OS: 9.7 months
O’Reilly et al. 2019 [122]	II (NCT02558894)	Anti PD-L1 antibody (Durvalumab) + Anti CTLA4 antibody (Tremelimumab)	65	No objective response
Renouf et al. 2020 [123]	II (NCT02879318)	Gembitabine + Nabpaclitaxel ± Durvalumab + Tremelimumab	180	No objective response

GVAX: granulocyte-macrophage colony-stimulating factor (GM-CSF) gene-transfected tumor cell vaccine; OS: overall survival.

## 6. Conclusions

The immune landscape of human PDAC is inextricably linked to the abundant desmoplastic stroma. That dense extracellular matrix contributes to the poor immunogenicity of PDAC, which impedes effector T-cell infiltration and thus promotes an immunosuppressive microenvironment.

Moreover, other extrinsic factors, especially the PDAC microbiome, may impact immune TME in human PDAC through immunomodulation.

There is an urgent need to better understand the interactions between tumor cells, immune cells, microbiome, and stromal components of pancreatic cancer. Further refining our knowledge of those interactions will be crucial to improve therapeutic strategies for human PDAC in the future.

## Figures and Tables

**Table 1:** Studies assessing CD3+ T cells expression in pancreatic ductal adenocarcinoma and the correlation with survival data.

Reference	Number of Patients	Statistical Significance
Ryschich et al. 2005 [44]	46	NS
Tewari et al. 2013 [45]	81	Significantly associated with improved OS
Chen et al. 2014 [46]	63	Significantly associated with improved OS
Helm et al. 2014 [47]	42	NS
Zhou et al. 2015 [51]	158	Significantly associated with improved OS (RR = 0.611)
Hwang et al. 2016 [52]	30	NS
Lundgren et al. 2016 [53]	175	Significantly associated with improved OS (HR = 0.42)
Lohneis et al. 2017 [56]	165	NS
Ino et al. 2019 [58]	241	Significantly associated with improved OS and DFS
Miksch et al. 2019 [59]	57	Significantly associated with improved OS and DFS

DFS: disease-free survival; HR: hazard ratio; NS: not significant; OS: overall survival; RR: relative risk.

**Table 2:** Studies assessing CD4 and CD8 T-cell expression in pancreatic ductal adenocarcinoma and the correlation with survival data.

Reference	TILs	Number of Patients	Statistical Significance
Fukunaga et al. 2004 [39]	CD4 + CD8	80	CD4/8 (+/+) significantly associated with better OS 5 years OS CD4/8 (+/+) = 48.4% versus 4.6% in CD4/8 (−/−) patients
Ryschich et al. 2005 [44]	CD4 + CD8	46	NS
Tewari et al. 2013 [45]	CD8	81	NS
Chen et al. 2014 [46]	CD8	63	NS
Tang et al. 2014 [68]	CD8	160	CD8^+^ cells significantly associated with better OS (HR = 0.56)
Castino et al. 2015 [48]	CD8	104	NS
Karakhanova et al. 2015 [69]	CD4 + CD8	92	CD4/8 (+/+) significantly associated with better DFS and OS
Liu et al. 2015 [71]	CD8	92	CD8^+^ cells significantly associated with better OS (low versus high CD8: mean 14.2 months versus 31.0 months)
Wartenberg et al. 2015 [50]	CD8	110	Reduce CD8^+^ cells are significantly associated with worse prognoses
Diana A et al. 2016 [62]	CD8	145	CD8+ cells significantly associated with better PFS (low versus high CD8: mean OS = 23.7 versus 33.8 months)
Hwang et al. 2016 [52]	CD4 + CD8	30	NS
Balachandran et al. 2017 [54]	CD8	166	CD8^+^ cells significantly associated with better OS
Carstens et al. 2013 [55]	CD4 + CD8	132	NS
Knudsen et al. 2017 [64]	CD8	109	NS
Lohneis et al. 2017 [56]	CD8	165	CD8^+^ cells significantly associated with better OS and DFS
Wang Z et al. 2017 [70]	CD4 + CD8	90	CD4/8 (+/+) significantly associated with better OSmedian OS CD4/8 (+/+) = 28 months versus 15 months in CD4/8 (−/−) patients
Nizri et al. 2018 [65]	CD8	66	CD8^+^ cells significantly associated with better OS(low versus high CD8: mean OS = 24.3 versus 36.8 months)
Pu et al. 2018 [66]	CD8	90	NS
Sideras et al. 2018 [67]	CD8	148	CD8^+^ cells significantly associated with better OS
Tahkola et al. 2018 [57]	CD8	108	NS
Danilova et al. 2019 [61]	CD8	33	NS
Hou et al. 2019 [63]	CD8	86	CD8^+^ cells significantly associated with better OS(low versus high CD8: median OS = 10.9 versus 25.8 months)
Ino et al. 2019 [57]	CD4 + CD8	241	CD4/8 (+/+) significantly associated with better OS
Miksch et al. 2019 [58]	CD8	57	CD8^+^ cells significantly associated with better OS and DFS

DFS: disease-free survival; NS: not significant; OS: overall survival; PFS: progression-free survival; TILs: tumor-infiltrating lymphocytes.
